# Editorial: Innate immune cell therapy of cancer

**DOI:** 10.3389/fimmu.2022.1004415

**Published:** 2022-08-19

**Authors:** Natasha Khatwani, Rizwan Romee, Asha B. Pillai

**Affiliations:** ^1^ Department of Pediatrics, Stuart M. Miller School of Medicine, Miami, FL, United States; ^2^ Department of Microbiology & Immunology, Stuart M. Miller School of Medicine, Miami, FL, United States; ^3^ Sheila and David Fuente Program in Cancer Biology, University of Miami, Miami, FL, United States; ^4^ Program in Tumor Biology, Sylvester Comprehensive Cancer Center, University of Miami, Miami, FL, United States; ^5^ Division of Hematologic Neoplasia, Dana Farber Cancer Institute, Harvard Medical School, Boston, MA, United States

**Keywords:** innate immunity, immunotherapy, natural killer (Nk) cell, natural killer T (NKT) cells, gamma-delta (γ/δ) T lymphocytes, macrophage, CAR-NK cell, CAR-gamma delta T cells

According to data from clinicaltrials.gov, the past year has seen a 33% growth in research and development of innate cellular immunotherapies (1). As of April 2022, the majority of cellular therapy trials in oncology involve chimeric antigen receptor-transduced T cells (CAR-T) with roughly 82% of these being pre-clinical and phase I studies and only 0.4% (6 total) FDA-approved (1). Major limitations to these CAR-T approaches include: 1) “off-tumor” toxicities including cytokine release syndromes, 2) potential for graft-versus-host disease (GVHD) associated with allogeneic third-party (“off-the-shelf”) T cell sources, 3) suboptimal killing by fatigued or senescent autologous T cells, and 4) poor recovery and expansion of autologous T cells from heavily pre-treated cancer patients.

Owing to their lack of HLA-restriction and ability to regulate allo-responses (1, 2) while addressing several of the above limitations to standard adoptive cellular immunotherapies (ACIs), innate immune cells (NK, NKT, γδT, and myeloid cells) may present a safe, robust, and cost-effective “off-the-shelf” improvement over conventional CAR-T immunotherapies. This Research Topic focuses on innate immune cells as the next frontier in ACI by highlighting some recent pre-clinical and clinical advances in innate ACI platforms against various malignancies. In this regard, it is notable that CAR strategies were originally envisioned and designed by the Campana research group for innate immune targeting and activation (3).

In this edition, Rossi et al. overview key features of NK cells that make them promising ACIs. NK cells kill target cells by perforin-mediated cytolysis, antibody-dependent cell cytotoxicity (ADCC), and/or cytokine/chemokine release. While therapeutic NK cells are easily sourced from peripheral blood (PB), mature PB NK cells can be functionally heterogeneous with low persistence *in vivo* post-adoptive transfer. To circumvent this limitation, Goldenson et al. use induced pluripotent stem cells (iPSCs) to produce functionally homogenous NK cells that can be reliably engineered for enhanced targeting, anti-tumor function, and persistence. Karvouni et al. overview recent key advances in non-CAR and CAR-engineered NK cells in pre-clinical and clinical settings, including novel combinatorial strategies with anti-angiogenic agents, oncolytic virotherapy, and monoclonal antibodies.

What defines a robust killer cell? Barnes et al. review criteria to consider when selecting optimal NK cell populations for ACI. This article addresses the dual problems of functional heterogeneity across ACI donor sources and differences in NK subset immunobiology within a single donor, highlighting the need to develop a standardized approach to functionally characterize NK cells with robust anti-tumor activity using transcriptomics, epigenomics, and metabolomics. Given the plasticity of innate immune cells, methods are needed to closely and efficiently monitor phenotypic and functional states to identify subsets with robust anti-tumor potential prior to adoptive transfer as well as to track functional changes in the tumor microenvironment (TME) post-transfer. Iyer et al. highlight one method to simultaneously detect up to 60 parameters using Time-Of-Flight (CYTOF^®^) mass cytometry. The article is intended as a comprehensive overview of the mass cytometry workflow from panel design to data analysis, providing a primer for immunologists with limited expertise in the technique.

NK cells are routinely expanded *ex vivo* using continuous addition of IL-15, a cytokine essential for stimulating NK proliferation and effector functions. However, these cells are found in circulation for only a few weeks post-adoptive transfer. One outstanding question in the field is how to improve *in vivo* persistence of innate ACIs. Mishra et al. show that ADAM-17, a membrane bound metalloprotease that mediates the cleavage or “shedding” of several cell surface proteins, attenuates prolonged IL-15-induced NK cell proliferation. Upon the addition of a well-characterized anti-ADAM-17 blocking human mAb (MEDI3622), IL-15-induced NK cell expansion and proliferation significantly increased *in vitro* and for up to 3 weeks post- *in vivo* adoptive transfer. The authors underscore the potential role of ADAM-17 blockade in enhancing IL-15-induced NK cell *ex vivo* expansion and *in vivo* persistence, without the need for excessive cytokine addition which may lead to overstimulation and exhaustion.

γδT cells have also been recognized for their anti-tumor potential (4, 5). However,several unanswered basic questions limit their translation. Johanna et al. demonstrate the ability to engineer αβ T cells to express a Vγ5Vδ1 TCR (clone FE11), referred to as TEG011. The authors showed that CD4+ αβ T cells displayed enhanced anti-tumor cytotoxicity after being transduced with a CD8α-containing TEG011 construct, called TEG011_CD8α and persisted in the periphery of mice for up to 4 weeks post-transfer.

Finally, one of the major unmet needs in CAR-T based ACI is to effectively target and maintain cytotoxicity within solid tumors. Marofi et al. summarize the major developments in CAR-NK immunotherapies against a variety of solid tumors in both pre-clinical and clinical settings including neuroblastomas, gastrointestinal, breast, and ovarian cancers. Although CAR-NK cells have proven safe and feasible as “off-the-shelf” ACIs, the following barriers have yet to be addressed: (1) tumor heterogeneity; (2) tumor homing; and (3) persistence in the suppressive TME. To overcome these barriers, Sloas et al. proposed CAR-Macrophages (CAR-M). Macrophages and other myeloid cells can effectively home to tumors, navigate through the dense stroma, persist in the harsh TME and recruit various other immune cell populations to help alleviate targeting barriers due to heterogeneity. The success of macrophage and other myeloid-derived immunotherapies will depend on engineering of CAR-Ms, as well as in preventing them from being reprogrammed by the suppressive TME.

The reviews and original articles in this Research Topic are curated with an eye to provide both depth of understanding and breadth of overview of the existing and upcoming landscape of innate cellular therapy platforms for cancer, highlighting some key challenges in each area and novel approaches that may advance future developments in the field. Key areas of need beyond the scope of this Special Edition include the development of pre-therapy guidelines and standardized approaches for patients with specific cancer sub-types or strategies to determine the best personalized ACI approach for individual patients. Successful application of these immunotherapies will also require a more rigorous understanding of tumor immune environments and associated alterations following various ACI approaches. These areas are expected to be the focus of the next generation of preclinical studies and clinical trials.

## Author contributions

NK wrote the manuscript. RR and AP wrote and edited the manuscript. AP conceived the manuscript. All authors contributed to the article and approved the submitted version.

## Conflict of interest

The authors declare that the research was conducted in the absence of any commercial or financial relationships that could be construed as a potential conflict of interest.

## Publisher’s note

All claims expressed in this article are solely those of the authors and do not necessarily represent those of their affiliated organizations, or those of the publisher, the editors and the reviewers. Any product that may be evaluated in this article, or claim that may be made by its manufacturer, is not guaranteed or endorsed by the publisher.

## References

[1] Saez-Ibañez AR Upadhaya S Partridge T Shah M Correa D Campbell J. Landscape of cancer cell therapies: trends and real-world data. Nat Rev Drug Discov (2022). doi: 10.1038/d41573-022-00095-1 35650421

[2] Andrews K Hamers AAJ Sun X Neale G Verbist K Tedrick P. Expansion and CD2/CD3/CD28 stimulation enhance Th2 cytokine secretion of human invariant NKT cells with retained anti-tumor cytotoxicity. Cytotherapy (2020) 22(5):276–90. doi: 10.1016/j.jcyt.2020.01.011 PMC7666372 32238299

[3] Imai C Iwamoto S Campana D. Genetic modification of primary natural killer cells overcomes inhibitory signals and induces specific killing of leukemic cells. Blood (2005) 106(1):376–83. doi: 10.1182/blood-2004-12-4797 PMC1895123 15755898

[4] Rozenbaum M Meir A Aharony Y Itzhaki O Schachter J Bank I. Gamma-delta CAR-T cells show CAR-directed and independent activity against leukemia. Front Immunol (2020) 2:1347. doi: 10.3389/fimmu.2020.01347 PMC7343910 32714329

[5] Makkouk A Yang XC Barca T Lucas A Turkoz M Wong JTS. Off-the-shelf Vδ1 gamma delta T cells engineered with glypican-3 (GPC-3)-specific chimeric antigen receptor (CAR) and soluble IL-15 display robust antitumor efficacy against hepatocellular carcinoma. J Immunother Cancer (2021) 2021(12):e003441. doi: 10.1136/jitc-2021-003441 PMC8679077 34916256

